# Co-infections in Visceral Pentastomiasis, Democratic Republic of the Congo

**DOI:** 10.3201/eid2208.151895

**Published:** 2016-08

**Authors:** Dennis Tappe, Mihály Sulyok, Therese Riu, Lajos Rózsa, Imre Bodó, Christoph Schoen, Birgit Muntau, Gergely Babocsay, Richard Hardi

**Affiliations:** Bernhard Nocht Institute, Hamburg, Germany (D. Tappe, B. Muntau);; Eberhard Karls University Tübingen, Germany (M. Sulyok);; Hôpital Géneral de Reference de Kole, Kole, Democratic Republic of the Congo (T. Riu);; MTA-ELTE-MTM Ecology Research Group, Budapest, Hungary (L. Rózsa);; Emory University School of Medicine, Atlanta, Georgia, USA (I. Bodó);; University of Würzburg, Würzburg, Germany (C. Schoen);; Mátra Museum of the Hungarian Natural History Museum, Gyöngyös, Hungary (G. Babocsay);; St. Raphael Ophthalmological Center, Mbuji Mayi, Democratic Republic of the Congo (R. Hardi)

**Keywords:** Armillifer grandis, Armillifer armillatus, Raillietiella sp., pentastomiasis, co-infection, PCR, zoonoses, Democratic Republic of the Congo, snakes, bushmeat, parasites

## Abstract

Snakeborne *Armillifer* pentastomiasis is an emerging human parasitic infection in rural tropical areas where snake meat is eaten. After a series of severe ocular *A. grandis* larval infections and anecdotal abdominal infection in Sankuru District, Democratic Republic of the Congo, during 2014–2015, we systematically investigated possible pentastomid etiology in patients who underwent surgery in the region. Histologic and molecular analyses by established pentastomid 18S rDNA- and newly developed *Armillifer*-specific cytochrome oxidase PCRs revealed larval pentastomid lesions in 3.7% of patients. Some persons had *A. armillatus* and *A. grandis* co-infections. Another pentastomid larva, *Raillietiella* sp., was molecularly detected in 1 patient who had concomitant *A. grandis* and *A. armillatus* infection. The PCRs used were suitable for detecting pentastomid species even in highly necrotic tissues. Phylogenetic analyses of *Armillifer* cytochrome oxidase genes detected multiple local strains.

## Abstract 2

Results of PCR and histology indicate this infection is endemic to this country.

Snakeborne pentastomiasis, a parasitic zoonotic disease in rural tropical areas where snake meat is eaten (*1*,*2*), is caused by a unique group of crustacean-related parasites (*3*,*4*). Adult *Armillifer* pentastomids inhabit the respiratory tract of large snakes (final hosts), where they sexually reproduce, resulting in shedding of infective ova into the environment by snake feces or respiratory secretions (*5*,*6*). In natural intermediate hosts (rodents and small monkeys), and accidentally humans, larvae hatch in the gastrointestinal tract after ingestion of pentastomid eggs, leading to dissemination and, eventually, to encapsulation of the vermiform larvae in internal organs (most often abdominal or peritoneal organs [visceral pentastomiasis (*1*)]) or in the eye (ocular pentastomiasis (*7**,**8*)]). Visceral pentastomiasis is often asymptomatic and an incidental finding during surgery or autopsy, and pentastomid larvae occasionally might be seen on radiologic films (*1*). However, fatal cases caused by heavy infections have been described (*9*). 

Human infections are caused mainly by larvae of *A. armillatus,* which is distributed in West and Central Africa (*1*). *A. grandis*, which has drawn recent attention because of heavily symptomatic ocular infections (*7*,*8*), is prevalent in Central Africa (*1*). Two other species, *A. moniliformis* and *A. akgistrodontis*, are found in Asia (*1*,*10*,*11*). 

*Armillifer* infection in humans is diagnosed by parasitologic examination of excised complete larvae or by histologic and radiologic investigations (*1*). Genus and species are determined by counting the body annulations of completely recovered specimens, but radiology and histology enable only limited conclusions about genus and species, respectively. In most cases, *A. armillatus* has been assumed to be the etiologic agent (*12*). Molecular tools recently have been used successfully in human infections for species discrimination in immigrants from tropical areas (*2*) and local populations in Africa (*8*,*12*). The main risk factors for human pentastomiasis caused by snakeborne parasites are handling and eating snake products (*1*,*3*). No effective chemotherapeutic antiparasitic treatment has been established (*1*).

In the Sankuru District, Democratic Republic of the Congo (DRC), severe ocular infections caused by *A. grandis* recently have surfaced (*7*,*8*). The same species also was molecularly found in the region in an asymptomatic abdominal infection (*12*), indicating a widespread problem in this remote area. We therefore conducted a cross-sectional observational study of patients undergoing abdominal surgery to investigate in detail the etiology of abdominal cystic lesions for the presence of pentastomid larvae in the district by histology and immunohistologic and molecular methods. In addition, we surveyed local markets for pentastomid-infested snakes.

## Patients and Methods

### Patients and Study Location

During December 1, 2014–March 31, 2015, we investigated cystic or fibrous lesions found incidentally during abdominal surgery from patients at the Hospital of Kole, Kole, Sankuru District, DRC. The medical center serves an area of 9,840 km^2^ of mainly tropical rain forest. Most patients come from the Kutshu, Hindu, and Tétéla tribes, which inhabit a 200-km area around the hospital. Patients of both sexes >18 years of age were enrolled after providing written consent; oral consent was obtained from some because of illiteracy. The ethics committee of the St. Raphael Ophthalmological Center (Mbuji Mayi, DRC) approved the study (no. COR/CE/1-7/15). Samples excised from visceral surfaces or from the peritoneal cavity were fixed in 90% ethanol and transferred to the Bernhard Nocht Institute for Tropical Medicine (Hamburg, Germany) for histologic and molecular analysis for a presumptive pentastomid etiology.

We also surveyed local markets in the Kole area (3°27′37.24′′N, 22°26′33.13′′E) for snake meat. Large snakes offered by private hunters were analyzed directly for adult pentastomid infection of respiratory tissues. Pentastomids were extracted by using forceps and placed in 100% ethanol for later parasitologic and molecular examination.

### Tissue Analysis for Pentastomid Etiology

The ethanol-fixed tissue specimens were directly processed for PCRs targeting the nuclear pentastomid 18S rRNA gene (*3*,*8*) and the mitochondrial *Armillifer* cytochrome oxidase (*cox*) subunit I gene by newly designed PCRs (forward primer Arm-F 5′-AGCAATAATAGGAGGATTCGGGA-3′ and reverse primer Arm-R 5′-GGATGGTTGTAATRAAGTTGATTGAGC-3′) or were transferred to formalin and embedded in paraffin for histologic and immunohistochemical analyses, later also followed by PCR. For PCR of the ethanol-fixed specimens, soft cysts were treated with proteinase K, and calcified cysts were completely ground before digestion. DNA was extracted by using the DNeasy Blood & Tissue kit (QIAGEN, Hilden, Germany). PCRs from the formalin refixed and embedded specimens were conducted after proteinase K digestion and DNA extraction by using the QIAamp DNA FFPE Tissue kit (QIAGEN) from either 5-μm tissue sections or the whole paraffin block. The pentastomid 18S rDNA-PCRs were performed as previously described (*3*,*8*). For the *Armillifer cox* PCRs, 40 cycles with denaturation at 94°C for 40 s, annealing at 55°C for 40 s, and elongation at 72°C for 60 s were run. Positive PCR products (430 and 288 bp, respectively) were visualized by gel electrophoresis followed by sequencing and BLAST analysis (http://www.ncbi.nlm.nih.gov/blast).

Histologic examinations were conducted from hematoxylin and eosin and periodic acid-Schiff stained tissue sections. Immunochistochemical analyses for CD3 (rabbit monoclonal IgG, 1:400; Epitomics-Abcam, Cambridge, UK); CD20 (mouse monoclonal IgG, 1:150; Dako, Hamburg, Germany); and TFG-β (rabbit polyclonal IgG, 1:100; DCS, Hamburg, Germany) were performed according to the manufacturers’ instructions by using the 2-component AEC-2 detection chromogen kit (DCS; for CD3 and CD20) or the horseradish peroxidase/DAB supervision2 kit (DCS [for TGF-β]) for visualization after antigen retrieval with boiling in citric acid (pH 6.0) for 2–3 min. A light hematoxylin counterstain was used.

### Phylogenetic Analyses of Larval and Adult Pentastomids

We used the *cox* sequences of the respective pentastomid specimens recovered from patients and local snakes for a multiple sequence alignment with MUSCLE software (http://www.ebi.ac.uk/Tools/msa/muscle/). Poorly aligned and divergent positions were removed by using Gblocks with the following parameter settings: –t = d, –b1 = 14, –b2 = 14, –b3 = 8, –b4 = 5, –b5 = h. The final alignment consisted of 286 of the original 1,527 positions from 26 sequences. We used MEGA6 (http://www.megasoftware.net/) for subsequent substitution model estimation and for phylogenetic tree reconstruction.

## Results

**Figure 1:**
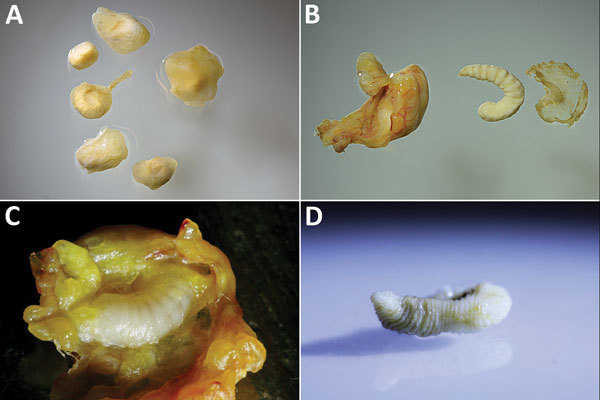
Resected cystic pentastomid lesions extracted from patients during abdominal surgery, Sankuru District, Democratic Republic of the Congo, 2014–2015. A) Six abdominal cysts resected from patient 3, who was found to be co-infected: 3 cysts each were *Armillifer grandis* and *A. armillatus* larvae, as determined by PCR. B) One of 2 resected *A. armillatus* cysts from patient 5. The fibrous capsule, the larva itself, and the parasite’s exuvia are shown. The larva has 20 annulations, morphologically consistent with the molecular result of *A. armillatus*. C) Resected and opened cyst from patient 5. The *A. armillatus* larva (as determined by PCR) is still embedded in its capsule. D) *A. grandis* larva from patient 2 with >25 annulations.

From 188 patients seen during the study period, we identified 7 (3.7%, of whom 4 were female) with incidental visceral fibrotic lesions, cystic lesions, or both (Table). Age (not determined for 4 patients) ranged from 35 to 42 years. All patients had undergone surgery for non–pentastomid-related conditions. A total of 23 lesions (1–6 individual cysts per patient) were resected. Multiple lesions (2 to 6) were resected in 5 (2.7%) of the 188 participants (Figure 1). All 7 patients with cystic lesions had pentastomid larvae as the underlying cause, as identified by pentastomid 18S rDNA PCR, *Armillifer cox* PCR, or both. In all but 3 lesions, at least 1 PCR was positive and gave a pentastomid sequencing result directly from the ethanol fixed specimens or from the formalin-fixed, paraffin-embedded tissue (tissue slice or whole block). In 2 of these 3 lesions, ovarial tissue was detected by histology (patient 6, cysts 2 and 3); in 1 necrotic pentastomid lesion, no positive PCR result was obtained (patient 1, cyst 4) (Table). 

**Table:** Patient characteristics and sequencing results of pentastomid 18S rDNA and *Armillifer*-specific cytochrome oxidase gene PCRs, Sankuru District, Democratic Republic of the Congo, 2014–2015*

Patient no.	Age, y/sex	Type of surgery	Location of pentastomid cyst	Sequencing result		Histology
				Pentastomid 18S rDNA-gene, 430 bp	*Armillifer* cytochrome oxidase subunit I-gene, 288 bp	
1†	NA/M	Laparotomy	Peritoneum	Cyst 1: *A. grandis*	*A. grandis*	Not done
				Cyst 2: negative	*A. armillatus*	Not done
				Cyst 3: *Raillietiella* sp.‡	Negative‡	Necrotic pentastomid
				Cyst 4: negative§	Negative§	Necrotic pentastomid
2†	35/F	Laparotomy	Peritoneum	Cyst 1: *A. grandis*	*A. grandis*	Not done
				Cyst 2: *A. armillatus*	*A. armillatus*	Not done
				Cyst 3: negative‡	*A. grandis*‡	Necrotic pentastomid
				Cyst 4: negative‡	*A. grandis*‡	Total necrosis
				Cyst 5: negative‡	*A. grandis*‡	Necrotic pentastomid
				Cyst 6: negative‡	*A. grandis*‡	Total necrosis
3†	NA/M	Laparotomy	Peritoneum	Cyst 1: *A. grandis*	*A. grandis*	Not done
				Cyst 2: *A. armillatus*	*A. armillatus*	Not done.
				Cyst 3: *A. grandis*	Negative	Not done
				Cyst 4: negative§	*A. grandis*§	Total necrosis
				Cyst 5: negative§	*A. armillatus*§	Total necrosis
				Cyst 6: negative‡	*A. armillatus*§	Total necrosis
4	NA/M	Hernioplasty	Peritoneum	Cyst 1: *A. armillatus*	*A. armillatus*	Not done
5	NA/F	Cesarean section	Omentum	Cyst 1: *A. armillatus*	*A. armillatus*	Not done
				Cyst 2: *A. armillatus*‡	*A. armillatus*‡	Necrotic pentastomid
6	36/F	Laparotomy, appendectomy	Left ovary	Cyst 1: *A. armillatus*	*A. armillatus*	Not done
				Cyst 2: not done	Not done	Ovarian cyst
				Cyst 3: not done	Not done	Ovarian cyst
7	42/F	Laparotomy, appendectomy	Omentum	Cyst 1: *A. armillatus*	*A. armillatus*	Not done

*NA, not available; negative, PCR negative or no positive sequence result. †Co-infected. ‡PCRs performed from paraffin block tissue slices. §Repeat PCRs performed from whole paraffin block, after PCRs from block slices were negative. Tissue materials from patient 1 (cysts 1, 2), patient 3 (cyst 3), and patient 7 (cyst 1) were ground, as the tissue was too hard to cut into pieces for PCR.

Pentastomids identified after sequencing (98%–100% homology) were *A. armillatus* larvae in 10 (43%) of 23 lesions, *A. grandis* larvae in 9 (39%), and *Raillietiella* sp. larva in 1 (0.04%). The *Raillietiella* sequence obtained was identical to 2 unspecified *Raillietiella* sp. GenBank entries (accession nos. EU370434 and AY744887). In total, we detected *A. armillatus* in all 7 patients. Co-infections with *A. armillatus* and *A. grandis* larvae were identified in 3 (43%) patients (patients 1–3); patient 1 had a triple pentastomid species infection, including a *Raillietiella* sp. larva.

**Figure 2:**
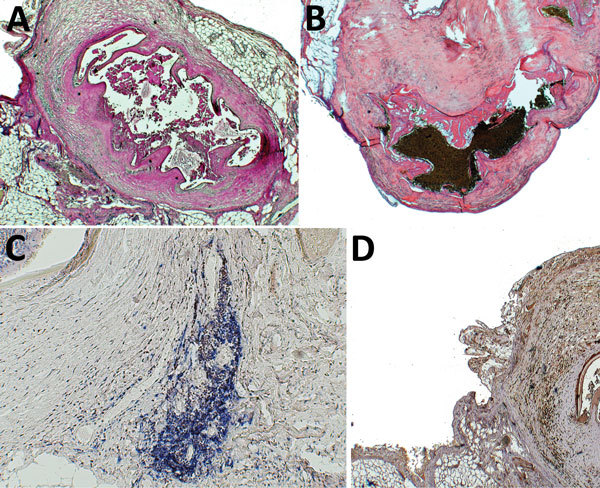
Histologic and immunohistochemical analyses of resected pentastomid lesions from patients in Sankuru District, Democratic Republic of the Congo, 2014–2015. A) Typical necrotic pentastomid lesion from patient 5. Internal structures of the larvae are decayed; only the directly surrounding exuvia following the annulated body of the parasite and the fibrous capsule are visible. This organism has been moleculary identified as *A. armillatus*. Periodic acid Schiff stain; original magnification ×2.5. B) Necrotic pentastomid lesion from patient 1, with a similar appearance. This organism has been molecularly identified as *Raillietiella* sp. Hematoxylin and eosin stain; original magnification ×2.5. C) Immunohistochemical staining of B cells (blue) and T cells (brown) surrounding the *A. armillatus* lesion from patient 5. The lymphocytes cluster locally adjacent to the lesion. Mouse monoclonal anti-CD20 and rabbit monoclonal anti-CD3 stain with hematoxylin counterstain; original magnification ×2.5. D) Immunohistochemical staining for TGF-β (brown) around an *A. armillatus* larva of patient 5. Immunoreactivity is seen surrounding the lesion and nonspecifically within the parasite larva itself. Rabbit polyclonal IgG with hematoxylin counterstain; original magnification ×2.5.

Histology was performed from 12 cysts (including the 2 diagnosed as ovarian cysts) resected from 5 patients. Necrotic pentastomids were identified in 5 cysts, from a total of 3 patients, and the decay-refractory shed exuvia surrounded by fibrosis was found (Figure 2, panels A, B). In 4 of these 5 necrotic pentastomid cysts, PCR and sequencing results were positive, even from tissue slices. We found total necrosis of histologically unknown etiology in an additional 5 cysts from 2 patients; in all of them, we obtained positive pentastomid PCR and sequencing results (Table).

Immunohistochemical analysis showed B cells and T cells clustering locally at the fibrous capsule of necrotic pentastomid lesions (Figure 2, panel C). Immunostaining for TGF-β showed a slight expression of TGF-β in fibrous tissue surrounding the larvae (Figure 2, panel D).

**Figure 3:**
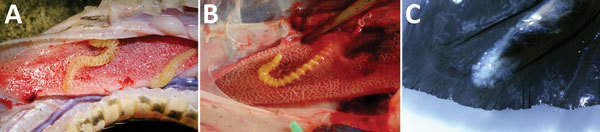
Adult *Armillifer* and *Raillietiella* parasites found in snakes at local markets in Kole, Sankuru District, Democratic Republic of the Congo, 2014–2015. A) Adult *A. grandis* in respiratory tract of a local rhinoceros viper (*Bitis nasicornis*). B) Adult *A. armillatus* in the lung of an African rock python (*Python sebae*) found in 2014, for comparison. Note the different annulation also between the adult stage of *A. grandis* and *A. armillatus*. C) Adult *Raillietiella* pentastomid in the lung of an African rock python. The rostral end of the parasite with central mouth and 2 pairs of perioral hooklets is shown.

Examination of 4 large snakes (2 Gaboon vipers [*Bitis gabonica*]*,* 1 rhinoceros viper [*B. nasicornis*], and 1 African rock python [*Python sebae*]) offered for consumption at markets near Kole during the study period showed pentastomid infections of the trachea and lungs and the mesenteric membrane. *A. grandis* was detected morphologically and molecularly (99%–100% sequence homology) in the 2 Gaboon vipers (8 and 10 parasites, respectively) and in the rhinoceros viper (10 parasites). The python harbored 6 adult *Raillietiella* sp. as identified by PCR (identical to the 2 unspecified *Raillietiella* sp. sequences in GenBank) (Figure 3).

**Figure 4:**
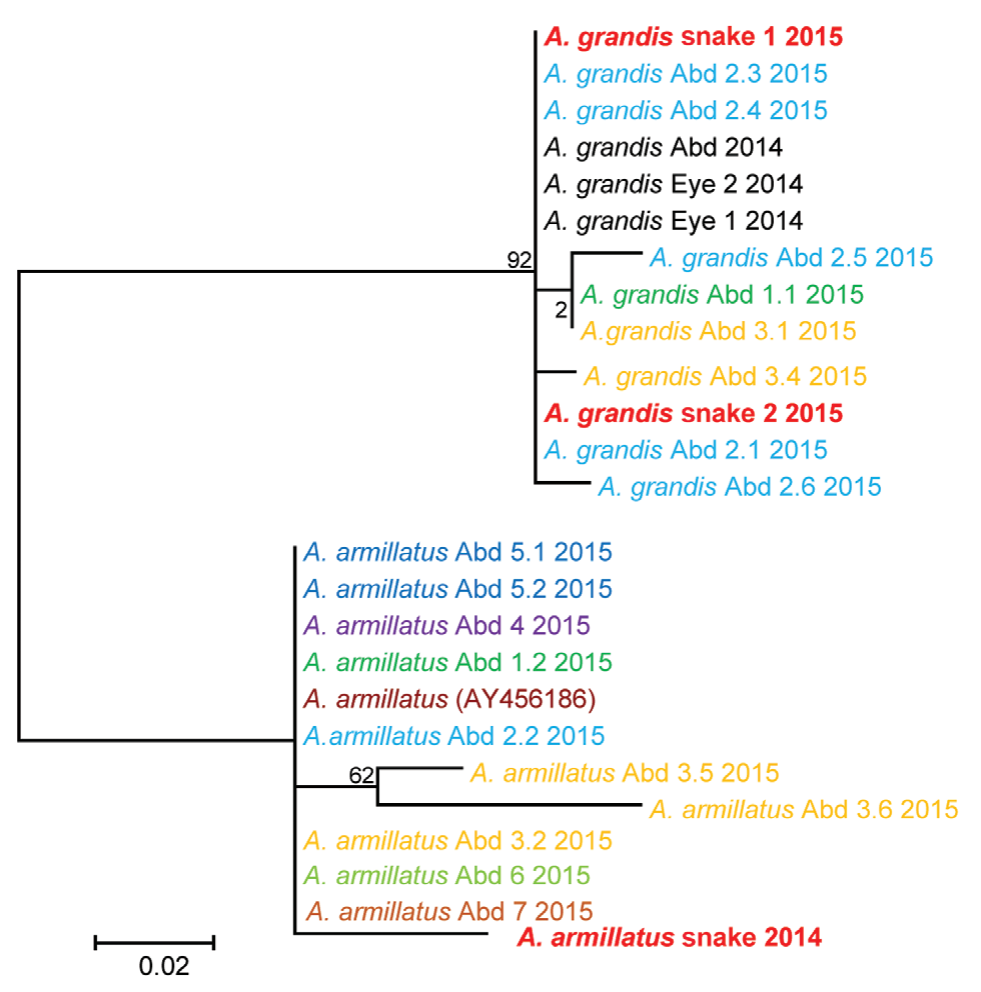
Molecular phylogenetic analysis of *Armillifer* spp. sequences obtained from humans and snakes in Sankuru District, Democratic Republic of the Congo, 2014–2015. Parasite cytochrome oxidase subunit I gene sequences from abdominal (Abd) surgery patient specimens (larval parasites) and from snake meats for sale at local markets (adult parasites) are shown. Human cases are numbered according to the patient and cyst numbers shown in the Table. Sequences obtained from the same patient share the same color. A GenBank reference sequence (*A. armillatus* AY456186) is included, as are sequences from the human abdominal and eye infections from the same region of the Democratic Republic of the Congo investigated in 2014 (*8*,*12*). The evolutionary history was inferred by using the maximum-likelihood method based on the Hasegawa-Kishino-Yano model. The tree with the highest log likelihood (−646.8057) is shown. The percentage of trees in which the associated taxa clustered together is shown next to the branches. Initial tree(s) for the heuristic search were obtained automatically by applying neighbor-joining and BioNJ algorithms to a matrix of pairwise distances estimated using the maximum composite likelihood approach, and then selecting the topology with superior log likelihood value. Codon positions included were 1st+2nd+3rd+Noncoding. *A. grandis* and *A. armillatus* sequences form their own respective branches. Scale bar indicates number of substitutions per site.

Phylogenetic analyses based on *cox* results obtained from patients’ samples and snake specimens showed sequence variations within the *A. grandis* and the *A. armillatus* branch, indicating the presence of multiple local parasite strains. The *Armillifer* sequences found in 1 patient did not all cluster directly together, suggesting multiple infection events or ingestion of multiple pentastomid species or strains at the same time. Some sequences from patients’ larval *Armillifer* parasites were highly similar to sequences from adult *Armillifer* parasites found in snakes (Figure 4). The *Raillietiella* sp. 18S rDNA sequence from the patient showed 100% nt identity with the sequence from the snake’s *Raillietiella*.

## Discussion

After reports of heavily symptomatic eye infections (*7*,*8*) and incidentally found asymptomatic abdominal infections (*12*) with pentastomids in the remote tropical and forested Sankuru District of DRC, we conducted a short-term systematic study that sheds light on visceral pentastomiasis as an emerging infectious disease in this area. The previously described series of ocular infections with *A. grandis* larvae in the region was believed to indicate a local problem (*8*) because ophthalmologic infections are severely symptomatic, whereas visceral (abdominal) cases usually go unnoticed (*1*). The prevalence of visceral pentastomiasis we detected was not totally surprising because an earlier report of 2 patients had suggested that emerging situation (*12*). However, multispecies pentastomid co-infections as described here were not anticipated. 

In this investigation, co-infection was detected molecularly by application of established pentastomid 18S rDNA PCRs and the newly developed *Armillifer*-specific *cox* PCRs. Positive PCR results were obtained from all but 1 resected pentastomid lesions, even from necrotic pentastomid cysts and necrotic lesions of histologically unidentifiable etiology. By histologic examination only, determining the pentastomid species, even in nonnecrotic lesions, is nearly impossible and would rely on counting the body annulations in arbitrary section planes. Molecular tools, as shown in this investigation, can facilitate diagnosis and are able to determine the causative organism to the species level, even in necrotic or calcified tissue samples.

We detected *A. armillatus* and *A. grandis* in roughly similar frequencies in the cysts we tested (43.5% and 39.1%, respectively). Whether the co-infections in 3 patients represent superinfections or simultaneous infection events remains unclear. The finding of highly similar sequences of *A. grandis* from local snakes and patients underscores local transmission, although the snake *A. armillatus* sequence was more divergent from patient *A. armillatus* sequences. *A. armillatus* is geographically more widely distributed; its preferred final hosts are mainly pythons (*1*,*3*,*13*), whereas vipers are usually the hosts for the more geographically restricted *A. grandis* (*1*). We did not find co-infected snakes, but co-infections occur in reptile hosts as well (*14*). 

Our finding of a third pentastomid species responsible for a human infection, *Raillietiella*, was completely unexpected. Earlier work in 1954 proposed that *R. hemidactyli*, a pentastomid using lizards as final hosts and coprophageous insects, such as cockroaches, as intermediate hosts, might cause a dermatologic condition similar to creeping eruption in Vietnam (*15*). The authors described the syndrome in 3 patients who had eaten live lizards, similar to a report from 1952 (*16*). However, *Raillietiella* was not proven as the causative agent in the reported disease (and human infection with this parasite in general) because the parasite (adult or larval) was not found in the skin; speculation was based on the finding of local lizards harboring this parasite (*15*). This investigation confirms that *Raillietiella* can cause human infection, similar to *Armillifer* parasites. We also showed that snake meat consumption might factor into transmission of this pentastomid parasite because a *Raillietiella* sp. was found in a local snake in the district with identical 18S rDNA sequences to those from the human infection. In contrast, the consumption of lizards, a reptile host in which *Raillietiell*a is common, is not a habit in this region.

In the human host, encapsulated pentastomid larvae can live for a few years, after which they calcify (which might be seen as C-shaped calcifications on radiology films [*1*]). Dying larvae are thought to release antigens, provoking an immune reaction (*1*). We detected an accumulation of B cells and T cells around the disintegrating pentastomid lesions. TGF-β, a fibrosis-inducing cytokine, was expressed in the fibrous capsule surrounding the parasite lesion. Because patients sometimes harbor hundreds of larvae without overt clinical symptoms (*1*), the host–parasite interface and possibly locally produced host cytokines or factors released by living pentastomids deserve future research attention. A local or systemic immunosuppression induced by secretion of soluble factors by pentastomid larvae might be possible.

The patients in our study regularly ate snake meat, as is the habit in this region. At local markets, we found adult *A. grandis* and *A. armillatus* parasites in snake meat on several occasions and once adult *Raillietiella* parasites. The presence of adult pentastomids in snakes is well known to the local population. The local residents believe that *Armillifer* pentastomids are internal structures of snakes, “spiral springs,” that help in the serpent’s locomotion. Residents are unaware of the infective nature of these structures and, during consumption of undercooked snake meat, spit out adult parasites. This habit seems highly risky, leading to ingestion of pentastomid ova released during chewing. Simple hygiene instructions, such as removal of any parasites visible in slaughtered serpents, hand washing after handling snake meat, and proper cooking of snake meat, is likely to reduce the risk. 

Our study also draws attention to the medical consequences of increasing bushmeat consumption in the Congo basin (*17*). Extensive deforestation and reduced availability of birds and mammals have resulted in consumption of snake and other reptile meat (*8*). Although these phenomena are not limited to Sankuru District, the geographic extent and incidence of snakeborne pentastomiasis as an emerging infectious disease is likely to increase. Currently, only scarce and outdated information exists about the prevalence of human pentastomiasis: radiology studies in the Congo region in the 1950s (<1% prevalence [*18*]), in Nigeria in the 1990s (1.4% [*19*]), a serosurvey in Côte d'Ivoire in the 1980s (4.2% [*20*]); and autopsy studies in the Congo region in the 1910s and 1930s (12%–22.5% [*21**,**22*]), in Cameroon in the 1910s (7.8%–12.6% [*23**,**24*]), Nigeria in the 1960s (33% [*25*]), and in indigenous people in Malaysia in the 1960s (45.4% [*26*]).

In summary, we undertook a surgery-based study of patients in a region where snake meat consumption is widely practiced to investigate etiology of abdominal cystic lesions for the presence of pentastomid larvae. Seroprevalence studies using crude parasite antigens (ELISA and immunoblot [*3*]) in the affected region will follow. Our findings indicate that the rate of visceral pentastomiasis as an incidental finding during surgery indicates endemicity, most likely fueled by consumption of bushmeat and animal exploitation.
